# Trends in inequalities in childhood overweight and obesity prevalence: a repeat cross-sectional analysis of the Health Survey for England

**DOI:** 10.1136/archdischild-2023-325844

**Published:** 2024-01-23

**Authors:** Philip Broadbent, Yue Shen, Anna Pearce, Srinivasa Vittal Katikireddi

**Affiliations:** 1 University of Glasgow MRC/CSO Social and Public Health Sciences Unit, Glasgow, UK; 2 NHS Education for Scotland, Edinburgh, UK

**Keywords:** Obesity, Child Health, Epidemiology

## Abstract

**Objective:**

To examine trends in socio-economic and ethnic inequalities in childhood overweight and obesity in the England between 1995 and 2019 in survey data and to compare these to administrative data.

**Design:**

Observational repeated cross-sectional study using the Health Survey for England (HSE) and National Child Measurement Programme (NCMP).

**Outcome:**

Age and sex standardised overweight, obesity and overweight including obesity.

**Analysis:**

Inequalities assessed by parental education, family structure, ethnicity (binary non-white vs white) and area-level Index of Multiple Deprivation. Estimates stratified by age and sex. Trends compared against NCMP data (age 4–5 and 10–11 years).

**Results:**

Prevalence of childhood overweight including obesity increased from 26.0% in 1995 to 31.7% in 2019, with the highest and fastest growing levels in those aged 11–15 years, rising from 29.7% to 38.0%. Despite a plateau in overall childhood obesity since 2004, differences between groups demonstrated widening inequalities over time. Inequalities widened by area-level deprivation, household educational attainment, household structure and ethnicity driven primarily by increased prevalence among socioeconomically disadvantaged children. For example, the gap between children from households with no qualifications versus degree-level qualifications increased from −1.1% to 13.2%, and the gap between single-parent households and couple households increased from 0.5% to 5.3%. HSE trends in prevalence of childhood overweight and obesity by deprivation quintile were consistent with those in NCMP.

**Conclusion:**

Overall levels of child overweight and obesity increased between 1995 and 2004. Since then, increases in prevalence among less advantaged groups have driven widening of inequalities.

**WHAT IS ALREADY KNOWN ON THIS TOPIC**

- Childhood obesity is a growing public health concern in the UK, with increasing prevalence and substantial economic implications.
- Observed trends in obesity prevalence show disparities across various social groups, with children from black and some Asian ethnic groups showing higher prevalence of obesity.
- Research methodologies, such as the Health Survey for England and the National Child Measurement Programme (NCMP), offer different lenses to view obesity trends.

**WHAT THIS STUDY ADDS**

- This research provides a detailed analysis of the rising trend in childhood obesity in England, revealing widening disparities across socio-economic, gender and ethnic dimensions.
- The study corroborates the existence of socio-economic inequalities in childhood obesity, providing a comprehensive longitudinal view using Health Survey for England (HSE) data.
- The socio-economic inequalities identified in this study are corroborated by data from the NCMP.

**HOW THIS STUDY MIGHT AFFECT RESEARCH, PRACTICE OR POLICY**

- This research underscores the need to consider socio-economic factors in addressing childhood obesity, particularly tailored interventions to reduce inequalities in health outcomes in children.

## Introduction

Childhood obesity and overweight pose significant health risks.^1–3^ In the UK, prevalence is higher than in comparable countries, with over a third of children having overweight or obesity.^4 5^ The UK is projected to be Europe’s most obese country by 2030, with over 35% of adults living with obesity.^6^ Addressing this issue is a public health priority,^7^ posing substantial costs.^8 9^ In 2019/2020, over one million hospital admissions listed obesity as a factor,^10^ with direct NHS costs estimated at £6.1 billion annually.^5^ The World Obesity Atlas projects the annual global economic burden to reach $4.32 trillion (nearly 3% of global GDP) by 2035, comparable to COVID-19’s 2020 impact.^11^ Preventing and treating obesity in children and young people is therefore crucial.

Prior research demonstrates increases in childhood obesity prevalence over time; age-standardised global prevalence rose from 0.7% to 5.6% among girls and from 0.9% to 7.8% among boys between 1975 and 2016.^12^ Although various studies have investigated inequalities in obesity by socio-economic characteristics and ethnicity,^13–15^ few explore trends across multiple social inequality dimensions.

Understanding these evolving inequalities is crucial as obesity drivers may vary across social groups. The Health Survey for England (HSE) provides a repeat cross-sectional view of inequalities over time, making it a valuable resource for comprehensive analysis. Nevertheless, declining response rates raise concerns about representativeness and potential biases in HSE when assessing trends, especially in childhood obesity.^16^ Biases in population surveys, including non-response bias, could be exacerbated by factors including socioeconomic circumstances and health status, potentially distorting obesity estimates.^16–21^ Administrative datasets like England’s National Child Measurement Programme (NCMP) are relatively unaffected by response biases. NCMP provides detailed insights into childhood obesity trends by ethnicity and area-level deprivation. Data from 2006/2007 to 2019/2020 show only a modest increase in childhood obesity across time, but notable increases in inequality in obesity across different levels of deprivation.^22^ However, NCMP does not monitor other social factors like family structure and family-level socioeconomic circumstances.

Consequently, comparing obesity trends and inequalities between administrative data and survey data can help demonstrate potential biases. Comprehensive comparative analyses focusing on childhood obesity remain scant, leaving the magnitude and direction of potential biases uncertain.

This study has three aims:

1. Analyse trends in childhood overweight and obesity from 1995 to 2019, using HSE data.
2. Explore inequalities in childhood obesity by ethnicity, gender, parental education and family structure.
3. Compare trends in childhood overweight and obesity inequalities as captured by HSE and NCMP.

## Methods

### Datasets

#### Health Survey for England

The HSE commissioned by the Department of Health and accessible from UK Data Service has been described in detail previously.^23^ Since 1991, it has annually collected nationally and regionally representative cross-sectional data on children’s health, including height and weight measurements from 1995 allowing for rigorous evaluation of long-term trends. In 2003, non-response weights were implemented to address rising non-response levels.^24^ Household serial number identification facilitated appending of socio-economic measures from adult to child data between 1995–2014. HSE covers community-dwelling households but may not capture certain populations like institutionalised children or those in non-traditional housing. For this analysis, we focused on children under 16, divided into three age brackets: 2–4, 5–10, and 11–15 years.

#### The National Child Measurement Programme

The NCMP is a public health initiative annually measuring heights and weights of over one million primary school children in years 1 (4–5 years) and 6 (10–11 years) since 2006. NCMP data reveal trends in childhood overweight and obesity, including regional demographics and temporal patterns. The data are reliable at national and regional levels but less so at the local authority level.^25^

NCMP data completeness improved from 80% in 2006 to 96% in 2019–2020, whereas response rates for HSE declined for children aged 0–15, from 67% in 2005 to 54.1% in 2019.^26^

### Outcome measurement

Body mass index (BMI: child’s weight (kg) by height squared (m^2^)) is used to determine overweight and obesity prevalence in children. International obesity task force (IOTF) criteria, extrapolating overweight and obesity thresholds from adult BMI levels of 25 and 30 kg/m^2^, respectively, were used for both HSE and NCMP data.^27^ While NCMP offers a breakdown by multiple weight categories (underweight, normal weight, overweight, obese and severely obese), HSE data lacked sufficient power to estimate these with precision. Consequently, we used three broader categories for all analyses: overweight, obese and overweight including obese. Precision estimates (95% CIs) were derived from the SE of the proportions.

### Measures of inequality

#### Household education attainment

The highest education level of individuals in the household (available between 1998 and 2014) was used to classify each child’s household into one of the four categories: degree/equivalent, General Certificate of Education Advanced Level (A level)/equivalent, General Certificate of Secondary Education (GCSE)/equivalent or no formal qualifications.

#### Family structure

Single-parent versus couple-parent families (1995–2014). We used STATA to manage household serial numbers, count household members and create a variable for children per household. Post 2015, the removal of household serial numbers from HSE limited trend analysis by household education and structure.

#### Ethnicity

Due to sample size limitations, participants were grouped as white or non-white.

#### Index of Multiple Deprivation

The Index of Multiple Deprivation (IMD) is a small area-based measure of multiple deprivation, including domains of income, employment, education, health, crime, barriers to housing and services, and living environment, providing a comprehensive representation of deprivation in England and is available at various geographic levels, including the lower super output area.^28^ Both HSE and NCMP use IMD as a measure of deprivation. IMD was introduced into HSE in 2003.

### Statistical analysis

Overweight and obesity trends in HSE were examined by estimating prevalence across all relevant years. Direct standardisation with Office of National Statistics mid-year estimates addressed age composition changes over time. Trends were plotted as line graphs overall and for each inequality dimension. We assessed potential non-response bias by comparing age group trends by deprivation in HSE to NCMP over years 2006–2019.

We analysed long-term inequality trends in IMD and household education using relative index of inequality (RII), which adjusts for annual sample size variations, enabling consistent comparisons across populations and time.^29^ We generated prevalence risk ratios with 95% CIs for IMD and household educational attainment using Poisson regression, adjusted for age and sex, with standard errors.^29 30^ Comparable trends in overweight, obesity and the RII by IMD for the NCMP data were derived using aggregate proportions categorised by age and multiple deprivation index.^31^

## Results

Our initial analytic sample from the HSE data comprised 65 253 individuals aged 2–15 years. Participants were excluded due to missing or incomplete data. Across the entire study period, 8670 patients were excluded, due to missing or not applicable ethnicity data (n=64) and missing or incomplete BMI data (n=8606). Therefore, 56 583 HSE dataset participants were included for analysis, with annual sample sizes varying between 1080 and 6529 (further exclusion details in online supplemental appendix).

10.1136/archdischild-2023-325844.supp1 Supplementary data

### Overall trends

**Figure 1:**
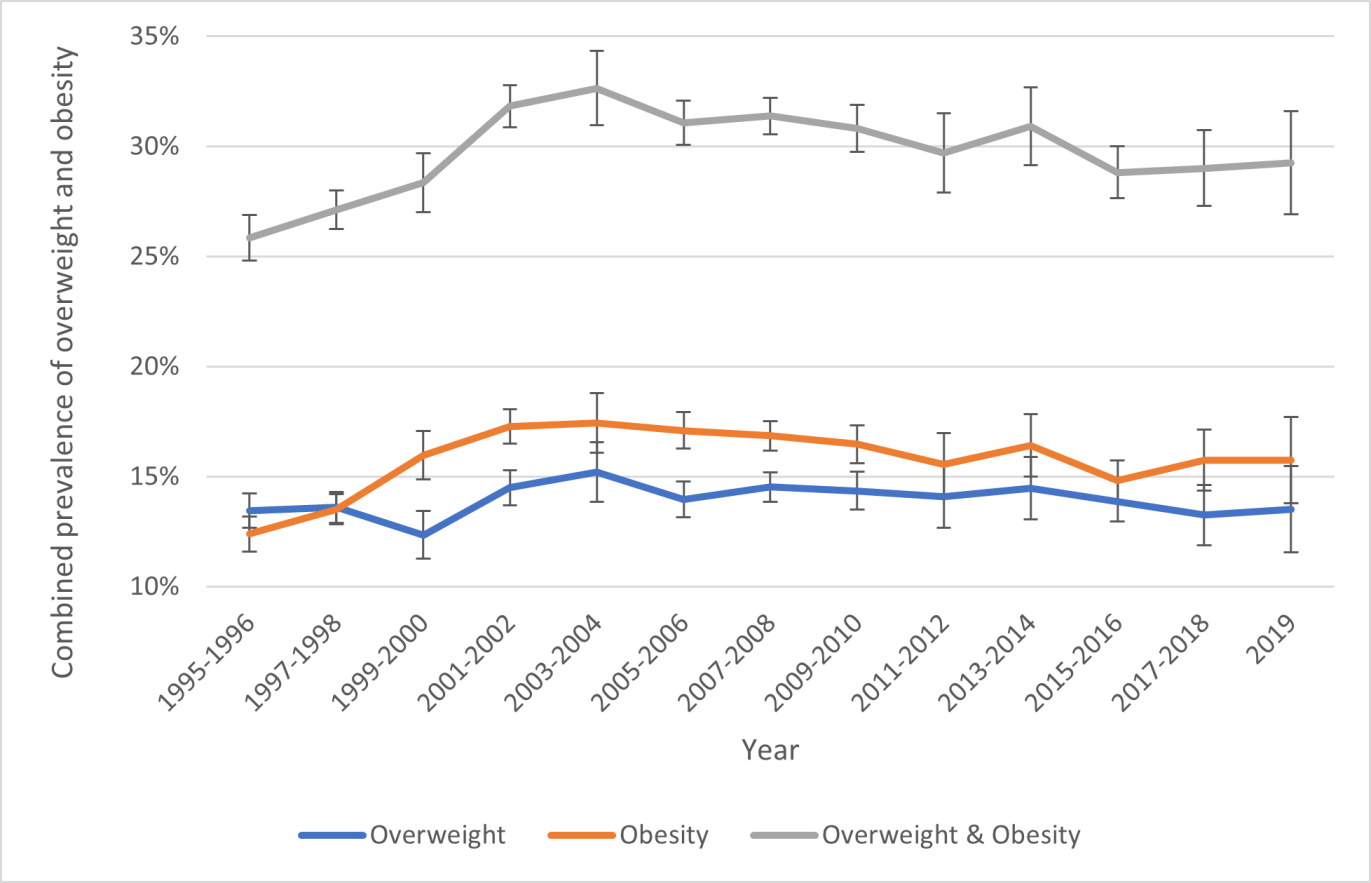
Age-standardised prevalence of childhood obesity in England (1995–2019), based on Health Survey for England data, with inverse probability weights applied.

Combined childhood overweight and obesity prevalence increased from 25.9% (95% CI: 24.8% to 26.9%) in 1995–1996 to 29.3% (95% CI: 26.9% to 31.6%) in 2019, with the highest at 32.7% in 2003–2004 (95% CI: 31.0% to 34.3%). This increase was primarily due to a rise in obesity prevalence from 12.4% (95% CI: 11.6% to 13.2%) in 1995–1996 to 15.7% (95% CI: 13.7% to 17.7%) in 2019. The highest prevalence was 17.4% (95% CI: 16.0% to 18.4%) in 2003–2004 whereafter prevalence appeared to plateau. Overweight prevalence fluctuated between 12.4% (95% CI: 11.4% to 13.4%) and 15.2% (95% CI 13.9% to 16.5%) with no clear trend (see figure 1).

Between 1995 and 2019, overweight and obesity prevalence in boys aged 11–15 years increased from 27.4% (95% CI: 25.0% to 29.9%) to 42.1% (95% CI: 35.1% to 49.1%), and in girls of the same age, prevalence increased from 28.3% (95% CI: 25.7% to 30.9%) in 1995 to 36.0% (95% CI: 29.4% to 42.6%) in 2019.

**Figure 2:**
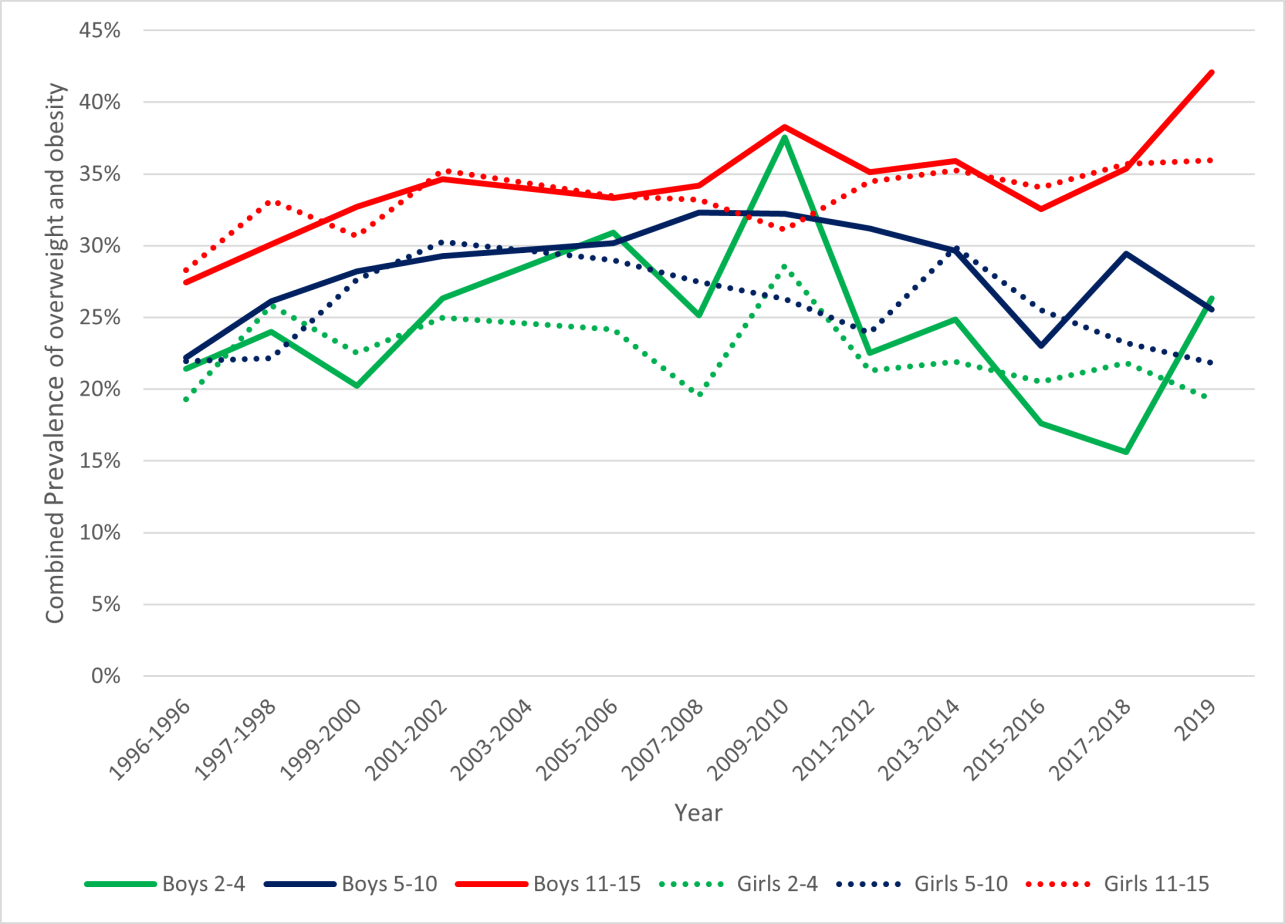
Age-standardised combined prevalence of overweight and obesity by age and gender, using Health Survey for England data (1995–2019) with inverse probability weights applied.

Conversely, prevalence in boys aged 2–4 years dropped from 30.9% (95% CI: 26.0% to 35.8%) in 2005–2006 to 26.3% (95% CI: 17.9% to 35.2%) in 2019. Prevalence also decreased among girls aged 5–10 years from 29.9% (95% CI: 26.0% to 33.8%) in 2013–2014 to 21.8% (95% CI: 16.5% to 27.2%) by 2019 (see figure 2). However, broad and overlapping CIs for these groups suggest caution in interpretation.

### Trends by socio-economic circumstances

**Figure 3:**
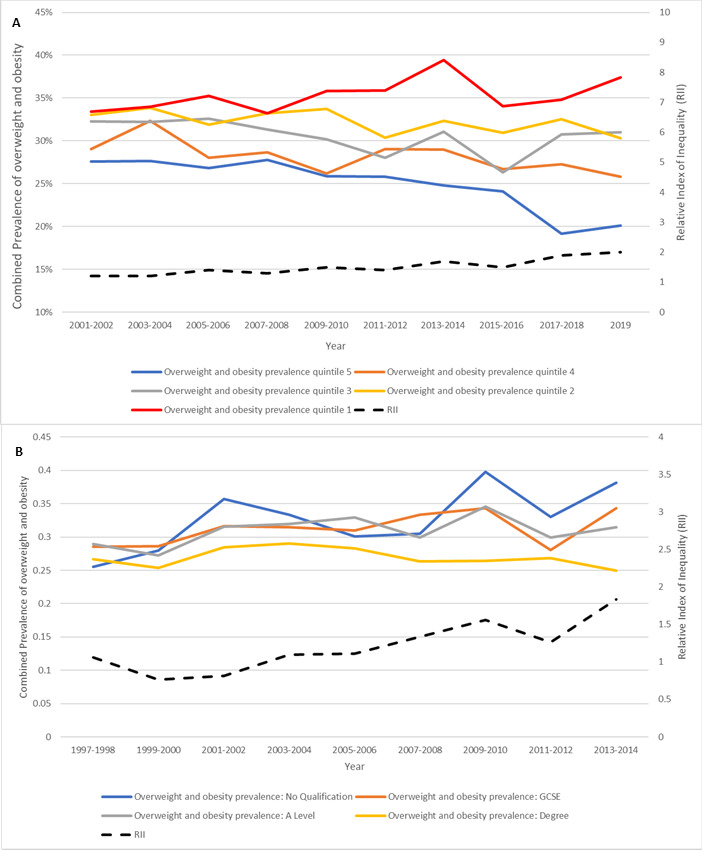
Age-standardised trends in combined prevalence of overweight and obesity by (A) deprivation quintile and Relative Index of Inequality (RII), 2001–2019, and (B) highest household education and RII, 1997–2014, analysed using Health Survey for England data with inverse probability weights applied. (A) Combined prevalence of overweight and obesity by deprivation quintile and RII, 2001–2019. (B) Overweight and obesity trends by highest household education and RII, 1997–2014.

Between 2001 and 2019, child obesity and overweight inequalities by deprivation widened, with RII rising from 1.2 (95% CI: 1.1 to 1.4) to 2.0 (95% CI: 1.6 to 2.4) (see figure 3A) Between 1997 and 2014, children in households with degree-educated adults generally had lower obesity rates compared with those with non-degree-educated adults and education-linked RII increased from 0.76 (95% CI: 0.69 to 0.84) indicating an inverse relationship between household education level and prevalence of overweight and obesity in 1999–2000 to 1.83 (95% CI: 1.8 to 1.9), indicating a reversal of the former trend, by 2014 (see figure 3B).

From 2003 onward, the increasing RII appears to be driven by stable prevalence of overweight and obesity in more advantaged groups and increasing prevalence among disadvantaged groups.

### Household structure

In 1995–1996, overweight and obesity prevalence was similar in children from single-parent families (26.3% (95% CI: 24.2% to 28.5%)) and couple families (25.8% (95% CI: 23.6% to 27.9%)). By 2015–2016, it rose to 34.0% (95% CI: 30.8% to 37.2%) in single-parent families compared with 28.7% (95% CI: 25.5% to 31.9%) in couple families (please see online supplemental appendix 1)

### Ethnicity

Initially, overweight or obesity was more prevalent in white children (26.0% (95% CI: 25.0% to 27.2%)) than in non-white children (24.4% (95% CI: 20.9% to 28.0%)). This trend reversed over time; by 2015–2016, prevalence was 25.9% (95% CI: 24.0% to 27.8%) in white children and 34.5% (95% CI: 30.6% to 38.4%) in non-white children, a pattern that persisted throughout the study. Detailed prevalence data by ethnicity and family structure are provided in online supplemental appendix.

### Comparison with NCMP

Despite HSE’s limitations, including smaller sample sizes and declining response rates compared with the stable NCMP data (details in online supplemental appendix), both datasets showed similar trends in childhood overweight and obesity.

**Figure 4:**
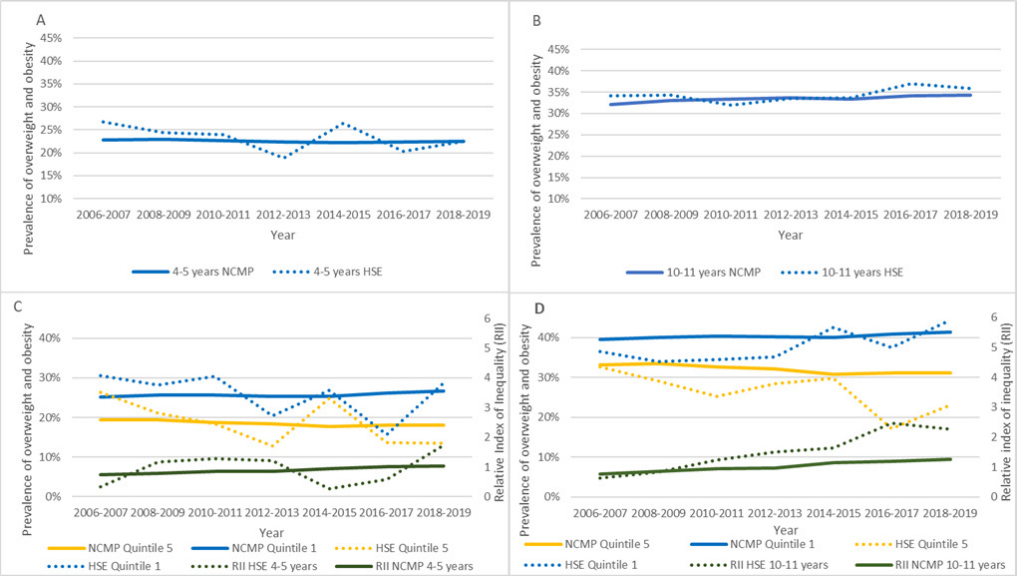
Age-standardised comparison of trends in prevalence of overweight and obesity in children aged 4–5 years (A) and 10–11 years (B), and by level of deprivation quintiles 1 and 5 among 4–5 year-olds (C) and 10–11 year-olds (D), using National Child Measurement Programme (NCMP) and Health Survey for England (HSE) data with inverse probability weights applied. RII, relative index of inequality.

Throughout the study period, both datasets demonstrate that overweight and obesity prevalence in 4–5 year-olds remained relatively stable. In contrast, prevalence among 10–11 year-olds increased in both NCMP and HSE (see figure 4A,B).

NCMP data consistently indicate higher overweight and obesity prevalence in children from the most deprived quintiles than less deprived counterparts, a gap that widens over time. This mirrors HSE findings (despite HSE’s more pronounced annual fluctuations, especially in the middle quintiles). Both datasets show a gradual increase in RII values, notably in the 10–11 age group, indicating increasing inequalities in overweight and obesity prevalence particularly among older children (see figure 4C,D).

Both sources therefore identified a rise in overweight and obesity prevalence among children over 10 years of age, a clear association between deprivation and increased weight status, and widening deprivation-based inequality over the study’s duration.

## Discussion

This study examined childhood overweight and obesity trends in England from 1995 to 2019, assessing various inequality dimensions. Findings indicate that despite a plateau in overweight and obesity prevalence since 2004, increases in certain groups drove widening inequalities.

The study found increases in overweight and obesity among boys and adolescents, with potential declines in girls and younger age groups. Marked socio-economic inequalities were evident: children from higher deprivation areas, non-white children and those from non-degree-educated or single-parent households saw rising prevalence of obesity and overweight. In contrast, prevalence in children from more advantaged backgrounds stayed relatively unchanged, contributing to increasing inequalities.

Widening inequalities by household level socio-economic circumstances in HSE were corroborated by NCMP data. Although HSE has limitations (eg, smaller sample size and variable response rate), both sources demonstrated increasing socio-economic inequalities in childhood overweight and obesity.

Existing literature demonstrates childhood obesity inequalities related to socio-economic status, race/ethnicity and other factors. Disadvantaged and ethnic minority children face higher risks of overweight or obesity.^14 32–35^ This social gradient persists over time, with evidence of growing inequalities. Between 1953 and 2015, obesity prevalence increased, with widening inequalities; initially, disadvantaged children were more likely to be underweight, but later became more prone to obesity.^36^ Recent Scottish data show persistent and widening area-level deprivation inequalities in childhood obesity.^37^ However, long-term trends across various inequality dimensions have been less explored. Addressing this, our study analysed 20 years of nationally and regionally representative HSE data, offering insights into children’s household circumstances and comparing these with routine national monitoring sources.

This study has several key strengths. Multiple dimensions of inequality, including different measures of socio-economic circumstances, were assessed, with consistency found when compared against NCMP data. IOTF thresholds, validated and commonly used in assessing child obesity and overweight, ensured reliable and comparable evaluation of trends across time. Age standardisation accounted for population composition variability, and inverse probability weighting reduced the impact of potential selection bias in the HSE.

However, several limitations must be considered. Binary classification of ethnicity restricted in-depth analysis of varying childhood obesity trends across diverse ethnic groups. Small sample sizes and variable response rates in HSE posed challenges in deriving robust conclusions, particularly for specific age groups over extended periods. Declining participation in cross-sectional studies influenced by factors like changes in communication technologies and survey scepticism^38^ can introduce biases.^16–18 20^ While analysis of NCMP data suggests these biases may not significantly distort our findings, caution is still warranted when interpreting single or year-pair results from HSE. Furthermore, constraints in sample size limited our ability to explore intersectionality, thus restricting our insights into the compound effects of different aspects of inequality.

We primarily aimed to monitor trends in inequalities in childhood overweight and obesity from a public health perspective rather than to establish causal relationships. Observed trends might be influenced by shifts in socio-economic groups' composition. For instance, an increasing proportion of children with degree-educated parents over time could amplify inequalities, as these families may have more resources and make better-informed health and dietary choices. By analysing data across deprivation quintiles and calculating RII in our analysis, we aimed to minimise the influence of such compositional changes.^29^ We employed a structured framework for descriptive epidemiological analysis, emphasising the quantification and characterisation of overweight and obesity trends within a specific population over time.^39^

Use of categorical BMI outcomes can obscure nuanced weight patterns occurring within BMI groups, especially so at more extreme BMI levels.^40–42^ Unfortunately, we did not have sufficient power to look at severe obesity. Finally, broad and overlapping precision measures in many estimates limit full confidence in the overall conclusions drawn from these data.

As public health issues, like childhood overweight and obesity, intensify, they typically manifest clearer social patterns and exacerbate inequalities,^43^ a trend evident in our study. This underscores the urgent need for consistent and robust public health policies to confront these growing disparities. Despite initiatives such as the introduction of the 2018 sugary drinks levy and the 2020 childhood obesity plan, concerns about the potential rollback of these measures post-COVID-19 have arisen.^44^ Addressing these challenges requires not only assessing the effectiveness of existing policies but also exploring innovative interventions tailored to the complex and evolving landscape of childhood obesity. Future research should explore the underlying mechanisms driving these trends, especially focusing on diverse and multifaceted inequalities. Our findings reinforce the importance of sustained policy efforts and research to mitigate health inequalities, essential for shaping a healthier, more equitable future for all children.

## Conclusion

This study demonstrated that stable overall trends in childhood overweight and obesity in England concealed deepening inequalities across deprivation, gender, family structure, ethnicity and parental education. These findings highlight the urgent need to prioritise understanding and addressing these inequalities as a public health imperative, given the serious health implications of childhood obesity. The current cost-of-living crisis threatens to further exacerbate these inequalities, impacting access to healthy foods, quality education, healthcare, safe environments and stable employment. Proactively tackling these social determinants is essential to curb the escalating impact of this crisis on childhood obesity and to narrow the health inequality gap.

## Data Availability

All data relevant to the study are included in the article or uploaded as supplementary information.
