# Supplementary material for: Trends in inequalities in childhood overweight and obesity prevalence: a repeat cross-sectional analysis of the Health Survey for England
Source: Arch Dis Child. 2024 Jan 23;109(3):233–9. doi: 10.1136/archdischild-2023-325844 (PMC10894838; doi:10.1136/archdischild-2023-325844)
Supplement: Supplementary data [file archdischild-2023-325844supp001.pdf]

Contents

Appendix I: Completion rate of Health Survey for England (HSE) and National Child Measurement Programme as a percentage of children eligible to participate 2

Appendix II: Proportion of children overweight, obese and overweight and obese as measured by Health Survey for England (1995-2019) and comparison with National Child Measurement Programme (2006-2019)........ 3

Boys........ 3

Girls 3

Boys & Girls........ 4

Age groups 5

2-4 years........ 5

5 -10 years........ 5

11-15 years........ 6

Age and gender........ 7

Socio-economic circumstances........ 9

Index of Multiple deprivation (Combined proportion of children overweight or obese) 9

Top Household Qualification (Combined proportion of children overweight or obese) 10

Household structure(Combined proportion of children overweight or obese) 11

Ethnicity (Combined proportion of children overweight or obese) 12

Comparisons between NCMP and HSE (Combined proportion of children overweight or obese) .. 13

By age group 13

By deprivation quintile........ 13

Appendix III STROBE flowchart demonstrating reasons for exclusion of participants 15

Appendix IV Data Availability throughout study........ 16

Appendix I: Completion rate of Health Survey for England (HSE) and National Child Measurement Programme as a percentage of children eligible to participate

Appendix Table 1: Table demonstrating completion rate of Health Survey for England (HSE) and National Child Measurement Programme as a percentage of children eligible to participate

	HSE	NCMP
2005	67%	
2006	66%	80%
2007	65%	88%
2008	63%	90%
2009	68%	91%
2010		93%
2011	65%	93%
2012	62%	93%
2013	62%	93%
2014	63%	94%
2015	62%	95%
2016	62%	95%
2017	63%	95%
2018	60%	95%
2019	58%	95%

Appendix II: Proportion of children overweight, obese and overweight and obese as measured by Health Survey for England (1995-2019) and comparison with National Child Measurement Programme (2006-2019)

Boys

Appendix Table 2: Age-standardised prevalence of childhood overweight and obesity among boys in England (1995-2019), Based on Health Survey for England data, with inverse probability weights applied

	Overweight (SE)	Obesity (SE)	Overweight and obesity (SE)
1995-1996	13.99% (0.59%)	12.00% (0.56%)	26.00% (0.75%)
1997-1998	13.87% (0.50%)	13.61% (0.49%)	27.49% (0.64%)
1999-2000	11.98% (0.68%)	16.51% (0.79%)	28.53% (0.96%)
2001-2002	14.26% (0.51%)	17.26% (0.56%)	31.56% (0.68%)
2003-2004	15.02% (0.93%)	17.63% (0.98%)	32.64% (1.22%)
2005-2006	14.01% (0.54%)	18.05% (0.61%)	32.08% (0.73%)
2007-2008	14.54% (0.45%)	17.57% (0.49%)	32.12% (0.60%)
2009-2010	15.01% (0.58%)	17.52% (0.62%)	32.56% (0.76%)
2011-2012	15.21% (1.02%)	16.00% (1.05%)	31.28% (1.32%)
2013-2014	14.14% (0.94%)	17.22% (1.03%)	31.38% (1.26%)
2015-2016	13.48% (0.64%)	15.72% (0.68%)	29.37% (0.85%)
2017-2018	13.53% (0.95%)	16.52% (1.01%)	30.07% (1.25%)
2019	12.20% (1.27%)	19.48% (1.49%)	31.69% (1.76%)

Girls

Appendix Table 3: Age-standardised prevalence of childhood overweight and obesity among girls in England (1995-2019), Based on Health Survey for England data, with inverse probability weights applied

	Overweight (SE)	Obesity (SE)	Overweight and obesity (SE)
1995-1996	12.91% (0.58%)	12.77% (0.58%)	25.69% (0.76%)
1997-1998	13.32% (0.49%)	13.42% (0.49%)	26.75% (0.64%)
1999-2000	12.72% (0.72%)	15.40% (0.78%)	28.17% (0.97%)
2001-2002	14.72% (0.52%)	17.28% (0.57%)	32.08% (0.69%)
2003-2004	15.37% (0.94%)	17.20% (0.97%)	32.62% (1.21%)
2005-2006	13.87% (0.55%)	16.12% (0.58%)	30.05% (0.72%)
2007-2008	14.51% (0.46%)	16.11% (0.48%)	30.63% (0.60%)
2009-2010	13.67% (0.57%)	15.38% (0.61%)	29.09% (0.76%)
2011-2012	12.95% (0.95%)	15.08% (1.03%)	28.11% (1.28%)
2013-2014	14.79% (0.98%)	15.63% (1.01%)	30.42% (1.27%)
2015-2016	14.21% (0.65%)	13.88% (0.64%)	28.20% (0.83%)
2017-2018	12.97% (0.93%)	14.97% (0.97%)	27.94% (1.23%)
2019	14.84% (1.36%)	11.99% (1.22%)	26.84% (1.68%)

Boys & Girls

Appendix Table 3: Age-standardised prevalence of childhood overweight and obesity (boys and girls) in England (1995-2019), Based on Health Survey for England data, with inverse probability weights applied

	Overweight (SE)	Obesity (SE)	Overweight and obesity (SE)
1995-1996	13.47% (0.42%)	12.39% (0.40%)	25.86% (0.53%)
1997-1998	13.60% (0.35%)	13.51% (0.35%)	27.12% (0.45%)
1999-2000	12.35% (0.50%)	15.96% (0.56%)	28.35% (0.68%)
2001-2002	14.49% (0.37%)	17.28% (0.40%)	31.82% (0.49%)
2003-2004	15.19% (0.66%)	17.44% (0.69%)	32.65% (0.86%)
2005-2006	13.96% (0.38%)	17.10% (0.42%)	31.07% (0.51%)
2007-2008	14.53% (0.32%)	16.85% (0.34%)	31.38% (0.42%)
2009-2010	14.35% (0.41%)	16.47% (0.44%)	30.82% (0.54%)
2011-2012	14.10% (0.70%)	15.55% (0.73%)	29.71% (0.92%)
2013-2014	14.47% (0.68%)	16.42% (0.72%)	30.91% (0.90%)
2015-2016	13.88% (0.46%)	14.82% (0.47%)	28.82% (0.60%)
2017-2018	13.25% (0.66%)	15.75% (0.70%)	29.01% (0.88%)
2019	13.52% (0.90%)	15.74% (1.00%)	29.26% (1.20%)

Appendix Figure 1: Line graph demonstrating age-standardised prevalence of childhood overweight, obesity and overweight and obesity among children in England (1995-2019), Based on Health Survey for England data, with inverse probability weights applied

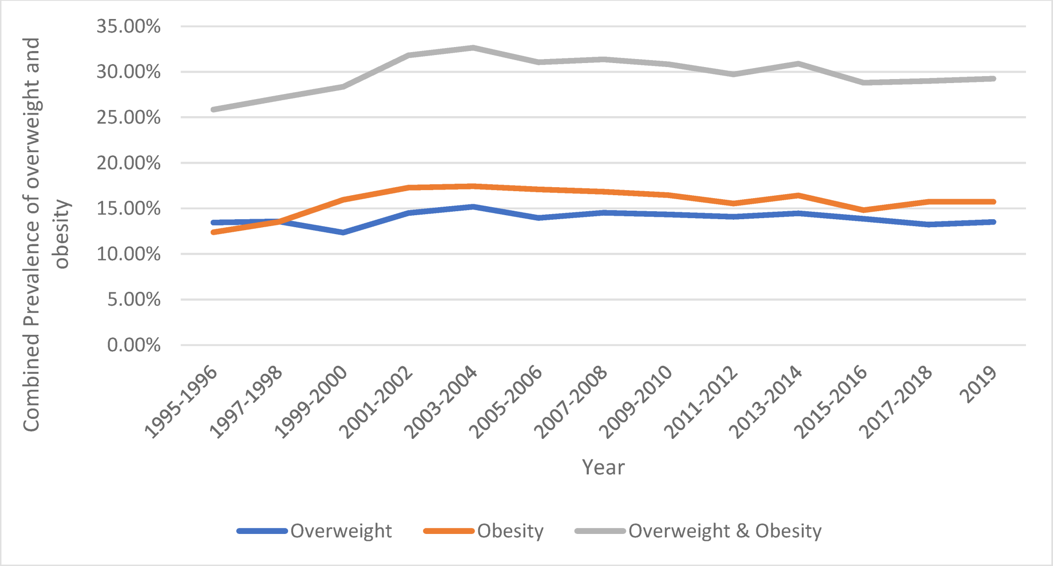

Age groups

2-4 years

Appendix Table 4: Age-standardised prevalence of childhood overweight and obesity among 2-4 year olds in England (1995-2019), Based on Health Survey for England data, with inverse probability weights applied

	Overweight (SE)	Obesity (SE)	Overweight and obesity (SE)
1995-1996	14.20% (0.90%)	9.88% (0.77%)	24.09% (1.10%)
1997-1998	14.61% (0.77%)	11.36% (0.70%)	25.97% (0.96%)
1999-2000	13.15% (1.11%)	10.07% (0.99%)	23.32% (1.39%)
2001-2002	15.08% (0.86%)	11.39% (0.76%)	26.73% (1.06%)
2003-2004	13.17% (1.43%)	13.01% (1.42%)	26.70% (1.87%)
2005-2006	14.40% (0.90%)	13.62% (0.88%)	28.09% (1.15%)
2007-2008	14.05% (0.72%)	11.92% (0.67%)	26.10% (0.91%)
2009-2010	16.00% (0.90%)	11.24% (0.77%)	27.25% (1.09%)
2011-2012	13.31% (1.22%)	9.09% (1.04%)	22.41% (1.50%)
2013-2014	14.45% (1.23%)	9.57% (1.02%)	24.69% (1.50%)
2015-2016	12.23% (0.94%)	11.12% (0.90%)	24.22% (1.23%)
2017-2018	14.05% (1.53%)	6.78% (1.11%)	20.88% (1.78%)
2019	14.19% (2.27%)	10.27% (1.99%)	24.47% (2.79%)

5 -10 years

Appendix Table 5: Age-standardised prevalence of childhood overweight and obesity among 5-10 year olds in England (1995-2019), Based on Health Survey for England data, with inverse probability weights applied

	Overweight (SE)	Obesity (SE)	Overweight and obesity (SE)
1995-1996	12.46% (0.59%)	11.10% (0.56%)	23.56% (0.76%)
1997-1998	12.54% (0.49%)	11.70% (0.48%)	24.26% (0.63%)
1999-2000	11.24% (0.70%)	15.45% (0.80%)	26.90% (0.98%)
2001-2002	13.41% (0.53%)	16.75% (0.58%)	30.24% (0.71%)
2003-2004	15.16% (0.98%)	15.89% (0.99%)	31.19% (1.27%)
2005-2006	12.66% (0.55%)	16.89% (0.62%)	29.57% (0.76%)
2007-2008	13.68% (0.47%)	15.97% (0.50%)	29.65% (0.62%)
2009-2010	12.90% (0.58%)	16.31% (0.64%)	29.22% (0.79%)
2011-2012	13.05% (0.98%)	14.83% (1.03%)	28.18% (1.30%)
2013-2014	13.78% (0.99%)	16.51% (1.06%)	30.30% (1.32%)
2015-2016	12.42% (0.64%)	13.62% (0.66%)	26.04% (0.85%)
2017-2018	11.74% (0.92%)	16.19% (1.05%)	28.00% (1.27%)
2019	12.38% (1.31%)	12.01% (1.24%)	24.39% (1.68%)

11-15 years

Appendix Table 6: Age-standardised prevalence of childhood overweight and obesity among 11-15 year olds in England (1995-2019), Based on Health Survey for England data, with inverse probability weights applied

	Overweight (SE)	Obesity (SE)	Overweight and obesity (SE)
1995-1996	24.09% (0.73%)	23.56% (0.74%)	29.71% (0.94%)
1997-1998	25.97% (0.62%)	24.26% (0.65%)	31.65% (0.81%)
1999-2000	23.32% (0.86%)	26.90% (0.99%)	32.07% (1.18%)
2001-2002	26.73% (0.61%)	30.24% (0.67%)	35.47% (0.81%)
2003-2004	26.70% (1.11%)	31.19% (1.18%)	36.60% (1.43%)
2005-2006	28.09% (0.64%)	29.57% (0.69%)	34.01% (0.84%)
2007-2008	26.10% (0.53%)	29.65% (0.57%)	35.06% (0.69%)
2009-2010	27.25% (0.68%)	29.22% (0.72%)	33.37% (0.89%)
2011-2012	22.41% (1.40%)	28.18% (1.51%)	36.35% (1.82%)
2013-2014	24.69% (1.30%)	30.30% (1.41%)	35.59% (1.70%)
2015-2016	24.22% (0.85%)	26.04% (0.89%)	34.70% (1.10%)
2017-2018	20.88% (1.17%)	28.00% (1.34%)	35.05% (1.59%)
2019	24.47% (1.58%)	24.39% (1.90%)	37.98% (2.18%)

Appendix Figure 2: Line graph demonstrating age-standardised prevalence of childhood overweight and obesity by age group in England (1995-2019), Based on Health Survey for England data, with inverse probability weights applied

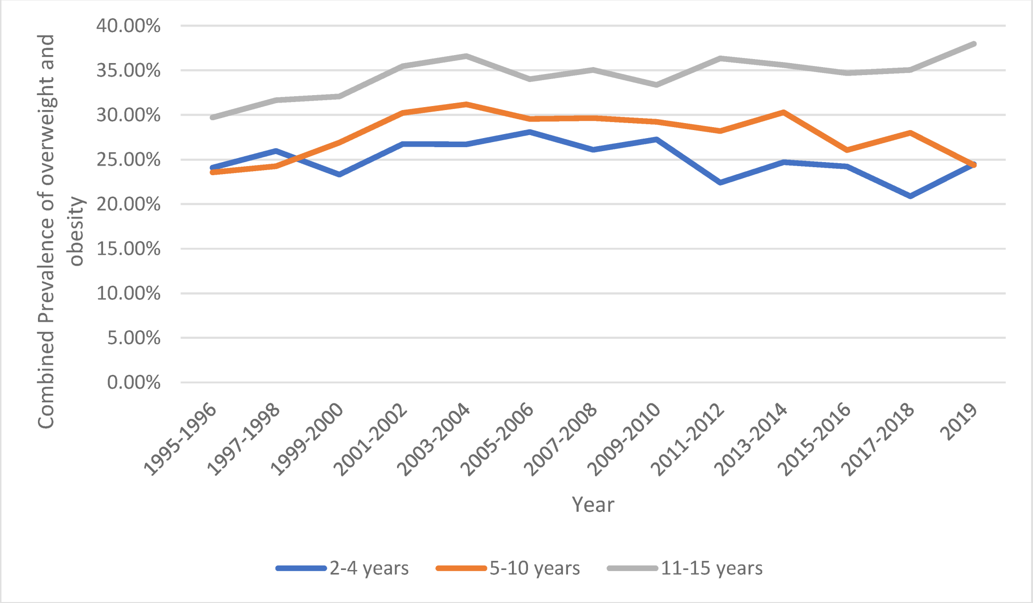

Age and gender

Appendix Table 7: Age-standardised prevalence of childhood overweight and obesity among children categorised by age and gender in England (1995-2019), Based on Health Survey for England data, with inverse probability weights applied

	Boys 2-4 (SE)	Boys 5-10	Boys 11-15	Girls 2-4	Girls 5-10	Girls 11-15
1995-1996	21.4% (1.4%)	22.2% (1.0%)	27.4% (1.3%)	19.3% (1.4%)	21.9% (1.0%)	28.3% (1.3%)
1997-1998	24.0% (1.3%)	26.1% (0.9%)	30.1% (1.1%)	25.8% (1.3%)	22.2% (0.9%)	33.2% (1.2%)
1999-2000	20.2% (1.6%)	28.2% (1.3%)	32.7% (1.5%)	22.5% (1.7%)	27.6% (1.28%)	30.7% (1.5%)
2001-2002	26.3% (1.5%)	29.3% (1.0%)	34.6% (1.2%)	25.0% (1.5%)	30.3% (1.0%)	35.3% (1.2%)
2003-2004	28.6% (1.6%)	29.7% (1.0%)	34.0% (1.1%)	24.6% (1.3%)	29.6% (1.1%)	34.4% (1.3%)
2005-2006	30.9% (2.5%)	30.2% (1.7%)	33.3% (1.8%)	24.2% (2.4%)	29.0% (1.6%)	33.4% (1.8%)
2007-2008	25.2% (2.4%)	32.3% (1.7%)	34.2% (1.9%)	19.5% (2.2%)	27.5% (1.7%)	33.2% (1.9%)
2009-2010	37.6% (3.3%)	32.2% (2.3%)	38.3% (2.4%)	28.6% (3.0%)	26.3% (2.2%)	31.1% (2.6%)
2011-2012	22.5% (2.2%)	31.2% (2.1%)	35.1% (2.6%)	21.3% (2.2%)	23.9% (1.8%)	34.5% (2.8%)
2013-2014	24.9% (2.2%)	29.6% (2.0%)	35.9% (2.5%)	21.9% (2.1%)	29.9% (2.0%)	35.3% (2.5%)
2015-2016	17.6% (2.3%)	23.0% (1.8%)	32.6% (2.4%)	20.5% (2.5%)	25.5% (1.86%)	34.1% (2.4%)
2017-2018	15.6% (2.4%)	29.4% (2.1%)	35.4% (2.4%)	21.8% (2.8%)	23.2% (1.8%)	35.7% (2.6%)
2019	26.3% (4.5%)	25.5% (2.6%)	42.1% (3.6%)	19.3% (3.7%)	21.8% (2.7%)	36.0% (3.4%)

Appendix Figure 3: Line graph demonstrating age-standardised prevalence of childhood overweight and obesity among children categorised by age and gender in England (1995-2019), Based on Health Survey for England data, with inverse probability weights applied

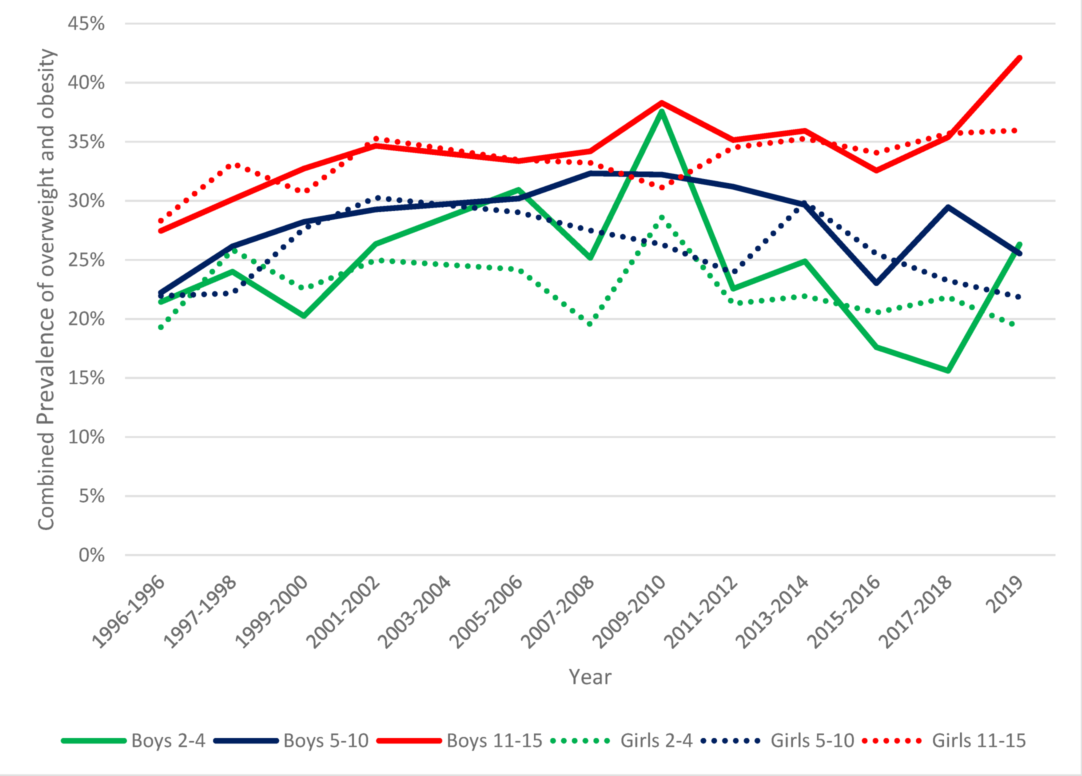

Socio-economic circumstances

Index of Multiple deprivation (Combined proportion of children overweight or obese)

Appendix Table 8: Age-standardised prevalence of childhood overweight and obesity among children categorised by deprivation quintile in England (2001-2019), Based on Health Survey for England data, with inverse probability weights applied

	2001-2002 (SE)	2003-2004 (SE)	2005-2006 (SE)	2007-2008 (SE)	2009-2010 (SE)	2011-2012 (SE)	2013-2014 (95% CI)	2015-2016 (SE)	2017-2018 (SE)	2019 (SE)
Least Deprived	27.58% (1.17%)	27.64% (1.87%)	26.80% 1.07%	27.77% (0.86%)	25.85% 1.06%	25.82% (1.81%)	24.80% 1.75%	24.07% (1.28%)	19.16% 1.70%	20.11% (2.46%)
Quintile 4	29.07% (1.21%)	32.35% (2.04%)	28.02% 1.11%	28.65% (0.94%)	26.19% 1.11%	29.03% (2.03%)	28.95% 2.04%	26.68% (1.36%)	27.26% 2.00%	25.80% (2.68%)
Quintile 3	32.29% (1.16%)	32.23% (1.95%)	32.59% 1.16%	31.31% (0.94%)	30.15% 1.20%	28.01% (2.08%)	31.06% 2.03%	26.29% (1.36%)	30.72% 2.14%	31.01% (3.00%)
Quintile 2	33.05% (1.07%)	33.88% (1.89%)	31.89% 1.14%	33.25% (0.97%)	33.74% 1.20%	30.37% (2.05%)	32.34% 2.03%	30.95% (1.41%)	32.55% 2.03%	30.30% (2.60%)
Most Deprived	33.44% (0.89%)	34.01% (1.73%)	35.27% 1.16%	33.24% (0.91%)	35.81% 1.19%	35.89% (2.25%)	39.46% 2.06%	34.07% (1.22%)	34.79% 1.86%	37.40% (2.70%)
RII	1.2 (0.08)	1.2 (0.13)	1.4 0.10	1.3 (0.07)	1.5 0.11	1.4 (0.18)	1.7 0.20	1.5 (0.13)	1.9 0.25	2.0 (0.35)

Appendix Figure 4: Line graph demonstrating age-standardised prevalence of childhood overweight and obesity among children categorised by deprivation quintile in England (2001-2019), Based on Health Survey for England data, with inverse probability weights applied

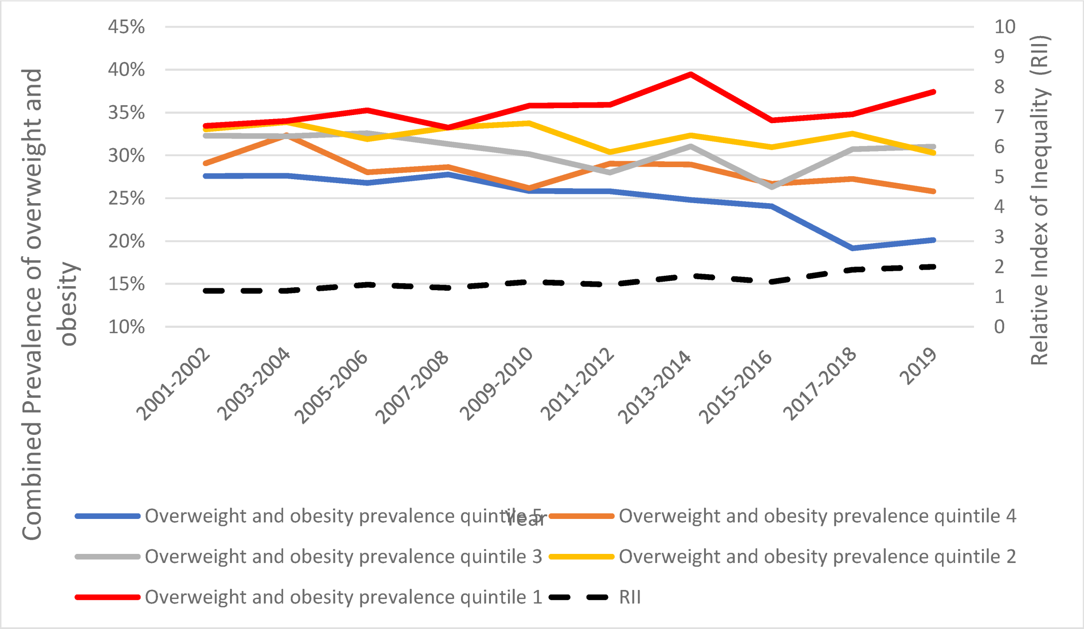

Top Household Qualification (Combined proportion of children overweight or obese)

Appendix Table 9: Age-standardised prevalence of childhood overweight and obesity among children categorised by top household qualification in England (1997-2016), Based on Health Survey for England data, with inverse probability weights applied

	No Qualification	GCSE	A Level	Degree	RII
1997-1998	25.51% (1.66%)	28.50% (1.03%)	28.95% (1.14%)	26.65% (1.38%)	1.06 (0.10)
1999-2000	27.94% (1.39%)	28.64% (1.30%)	27.20% (1.35%)	25.34% (1.59%)	0.76 (0.09)
2001-2002	35.68% (2.11%)	31.67% (1.08%)	31.52% (1.19%)	28.49% (1.42%)	0.82 (0.08)
2003-2004	33.37% (2.64%)	31.44% (1.49%)	31.99% (1.67%)	29.00% (1.74%)	1.10 (0.12)
2005-2006	30.06% (2.39%)	30.99% (1.42%)	32.93% (1.35%)	28.28% (1.38%)	1.11 (0.12)
2007-2008	30.52% (2.52%)	33.36% (1.52%)	29.92% (1.31%)	26.30% (1.36%)	1.34 (0.15)
2009-2010	39.80% (3.66%)	34.36% (1.96%)	34.56% (1.93%)	26.43% (1.66%)	1.56 (0.21)
2011-2012	33.01% (3.14%)	28.02% (1.95%)	29.93% (1.67%)	26.80% (1.45%)	1.26 (0.17)
2013-2014	38.15% (3.17%)	34.29% (2.04%)	31.48% (1.68%)	24.97% (1.37%)	1.83 (0.23)

Appendix Figure 5: Line graph demonstrating age-standardised prevalence of childhood overweight and obesity among children categorised by top household qualification in England (1997-2016), Based on Health Survey for England data, with inverse probability weights applied

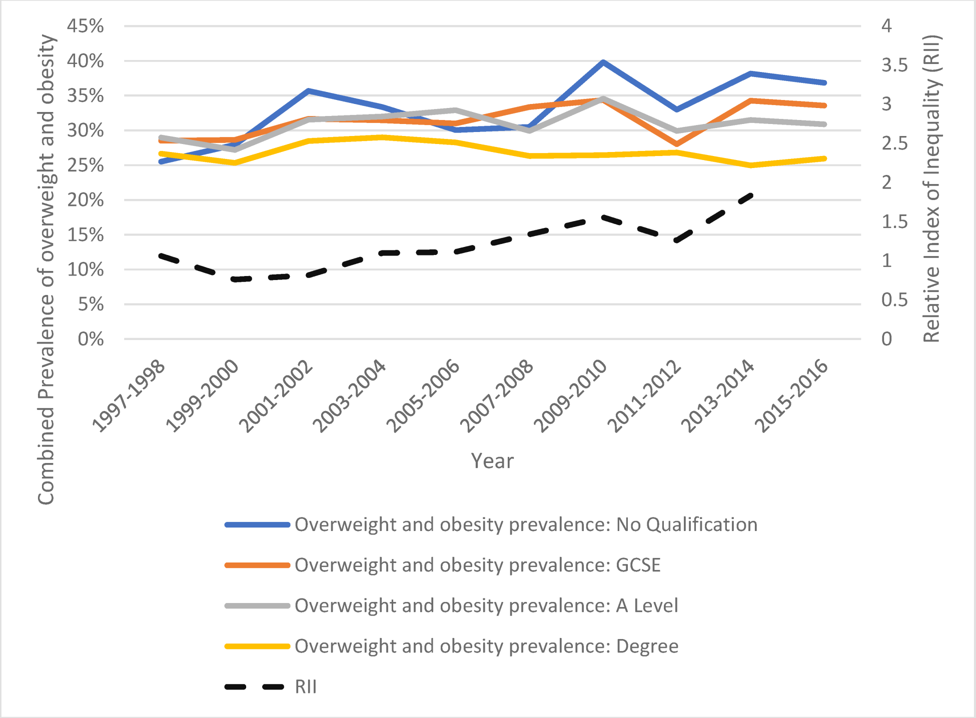

Household structure(Combined proportion of children overweight or obese)

Appendix Table 10: Age-standardised prevalence of childhood overweight and obesity among children categorised by household structure in England (1995-2014), Based on Health Survey for England data, with inverse probability weights applied

	Single Parent	Non Single Parent
1995-1996	26.31% (1.09%)	25.77% (1.09%)
1997-1998	30.00% (1.15%)	27.34% (1.15%)
1999-2000	27.96% (1.06%)	28.12% (1.06%)
2001-2002	33.08% (1.02%)	30.17% (1.02%)
2003-2004	31.30% (1.49%)	32.07% (1.49%)
2005-2006	31.34% (1.25%)	30.89% (1.25%)
2007-2008	32.52% (1.32%)	28.91% (1.32%)
2009-2010	35.66% (1.79%)	30.68% (1.79%)
2011-2012	30.48% (1.62%)	28.29% (1.62%)
2013-2014	33.96% (1.63%)	28.66% (1.63%)

Appendix Figure 6: Line graph demonstrating age-standardised prevalence of childhood overweight and obesity among children categorised by household structure in England (1995-2014), Based on Health Survey for England data, with inverse probability weights applied

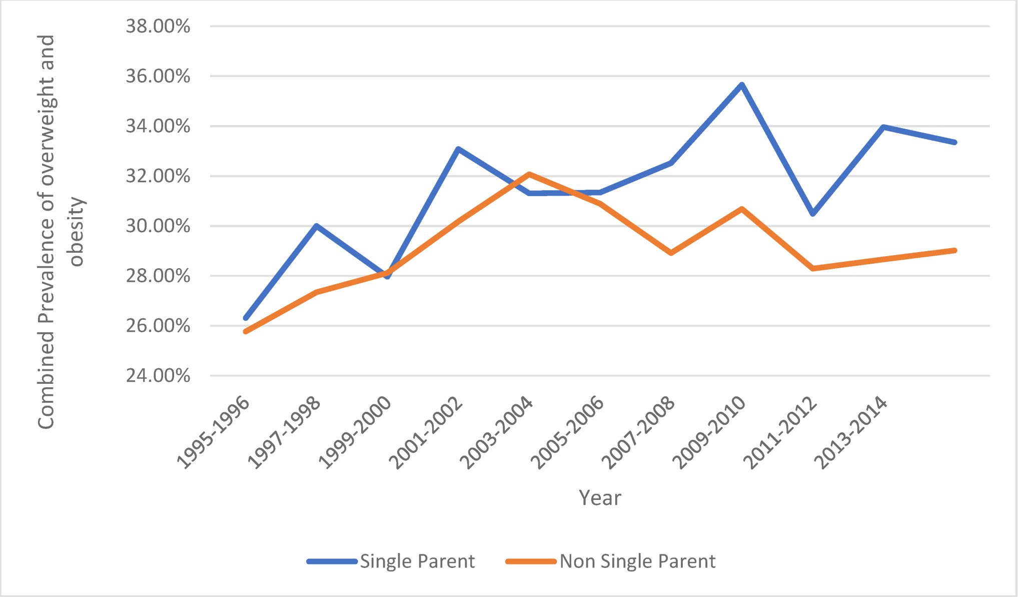

Ethnicity (Combined proportion of children overweight or obese)

Appendix Table 11: Age-standardised prevalence of childhood overweight and obesity among children categorised by Ethnicity in England (1995-2019), Based on Health Survey for England data, with inverse probability weights applied

	White (SE)	Non-White (SE)
1995-1996	26.05% (0.56%)	24.43% (1.82%)
1997-1998	27.33% (0.47%)	25.80% (1.63%)
1999-2000	28.00% (1.02%)	29.01% (0.93%)
2001-2002	31.24% (0.51%)	33.04% (1.38%)
2003-2004	31.11% (0.98%)	35.85% (1.96%)
2005-2006	30.40% (0.81%)	33.51% (2.00%)
2007-2008	29.55% (0.84%)	30.30% (2.24%)
2009-2010	31.33% (1.12%)	38.54% (3.72%)
2011-2012	27.73% (1.03%)	27.95% (3.40%)
2013-2014	29.86% (1.02%)	32.47% (2.48%)
2015-2016	25.89% (0.99%)	34.53% (1.99%)
2017-2018	28.55% (1.15%)	35.56% (2.19%)
2019	27.91% (1.44%)	33.43% (2.48%)

0

Appendix Figure 7: Line graph demonstrating age-standardised prevalence of childhood overweight and obesity among children categorised by Ethnicity in England (1995-2019), Based on Health Survey for England data, with inverse probability weights applied

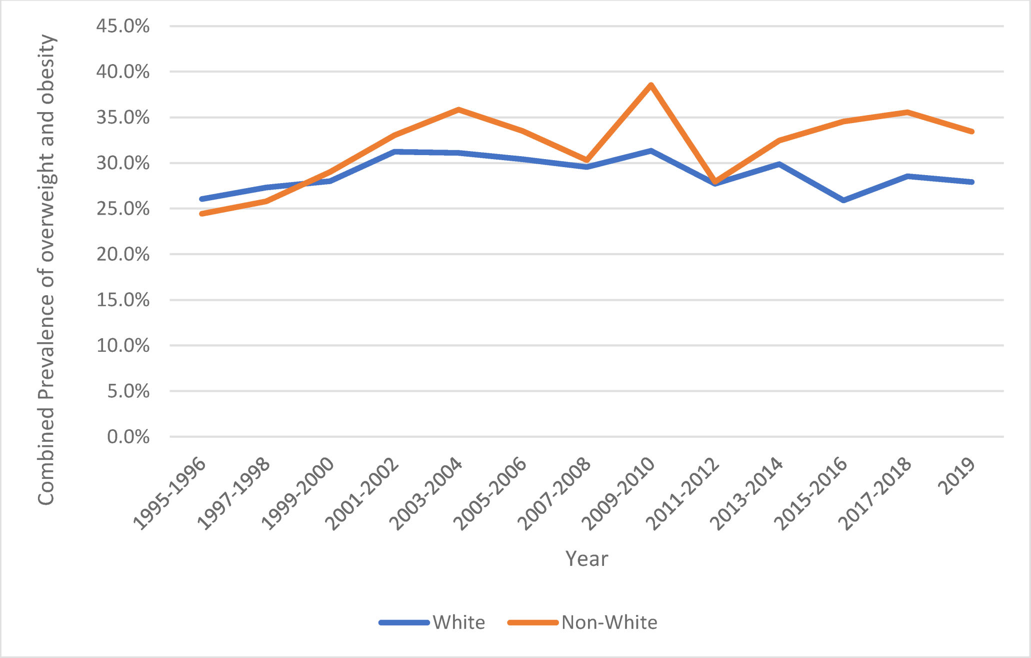

Comparisons between NCMP and HSE (Combined proportion of children overweight or obese)

Appendix Table 12: Age-standardised prevalence of childhood overweight and obesity among children categorised by age group in England (2006-2019), Comparing Health Survey for England (HSE) data and National Child Measurement Programme (NCMP) data

By age group

	4-5 years NCMP	4-5 years HSE	10-11 years NCMP	10-11 years HSE
2006-2007	23%	27%	32%	34%
2008-2009	23%	24%	33%	34%
2010-2011	23%	24%	33%	32%
2012-2013	22%	19%	34%	33%
2014-2015	22%	26%	33%	34%
2016-2017	22%	20%	34%	37%
2018-2019	22%	22%	34%	36%

By deprivation quintile

Reception (4-5 years)

Appendix Table 13: Age-standardised prevalence of childhood overweight and obesity among Reception age children (4-5 years old) categorised by deprivation quintile in England (2006-2019), Comparing Health Survey for England (HSE) data and National Child Measurement Programme (NCMP) data

	2006-2007	2008-2009	2010-2011	2012-2013	2014-2015	2016-2017	2018-2019
HSE 5	26.31%	21.03%	18.43%	12.77%	24.81%	13.73%	13.46%
HSE 4	27.63%	19.17%	20.57%	10.91%	26.61%	19.25%	20.39%
HSE 3	23.22%	23.01%	26.30%	25.09%	24.81%	28.34%	14.53%
HSE 2	25.98%	28.50%	22.33%	19.89%	27.69%	26.95%	24.57%
HSE 1	30.51%	28.12%	30.40%	20.50%	26.92%	15.68%	28.70%
	2006-2007	2008-2009	2010-2011	2012-2013	2014-2015	2016-2017	2018-2019
NCMP 5	19.40%	19.40%	18.80%	18.40%	17.70%	18.00%	18.10%
NCMP 4	21.10%	21.20%	20.70%	20.50%	19.80%	20.20%	20.30%
NCMP 3	22.30%	22.70%	22.30%	22.00%	21.60%	21.90%	22.30%
NCMP 2	24.40%	24.40%	23.90%	23.70%	23.50%	23.90%	24.00%
NCMP 1	25.10%	25.70%	25.70%	25.40%	25.30%	26.20%	26.70%

Year 6 (10-11 years)

Appendix Table 14: Age-standardised prevalence of childhood overweight and obesity among Year 6 age children (10-11 years old) categorised by deprivation quintile in England (2006-2019), Comparing Health Survey for England (HSE) data and National Child Measurement Programme (NCMP) data

	2006-2007	2008-2009	2010-2011	2012-2013	2014-2015	2016-2017	2018-2019
HSE 5	32.56%	29.15%	25.30%	28.40%	29.72%	17.16%	23.15%
HSE 4	31.49%	32.33%	33.21%	25.92%	29.07%	25.24%	35.97%
HSE 3	33.15%	36.40%	28.69%	33.47%	28.87%	30.34%	37.23%
HSE 2	36.11%	39.62%	39.69%	42.43%	36.17%	34.13%	38.79%
HSE 1	36.55%	33.96%	34.48%	35.13%	42.61%	37.43%	44.45%
	2006-2007	2008-2009	2010-2011	2012-2013	2014-2015	2016-2017	2018-2019
NCMP 5	33.13%	33.40%	32.67%	32.15%	30.86%	31.09%	31.15%
NCMP 4	35.70%	35.70%	35.27%	34.88%	33.82%	34.18%	33.93%
NCMP 3	36.79%	37.35%	36.94%	36.44%	35.92%	36.35%	36.51%
NCMP 2	38.12%	39.24%	38.69%	38.43%	38.32%	38.62%	38.60%
NCMP 1	39.49%	40.09%	40.36%	40.12%	40.03%	40.82%	41.34%

Relative index of Inequality by deprivation comparing HSE and NCMP data

Appendix Table 15: Relative index of inequality by deprivation among children categorised in England by age group (2006-2019), Comparing Health Survey for England (HSE) data and National Child Measurement Programme (NCMP) data

	2006-2007	2008-2009	2010-2011	2012-2013	2014-2015	2016-2017	2018-2019
HSE 4-5 years	0.3375	1.1755	1.285	1.222	0.265	0.58	1.733
NCMP 4-5 years	0.735	0.79	0.85	0.86	0.945	1.005	1.045
HSE 10-11 years	0.63	0.8455	1.242	1.4985	1.644	2.4715	2.271
NCMP 10-11 years	0.757	0.846	0.94	0.9745	1.142	1.195	1.2525

Appendix III STROBE flowchart demonstrating reasons for exclusion of participants

Appendix Figure 8: STROBE flowchart demonstrating reasons for exclusion of participants from HSE data

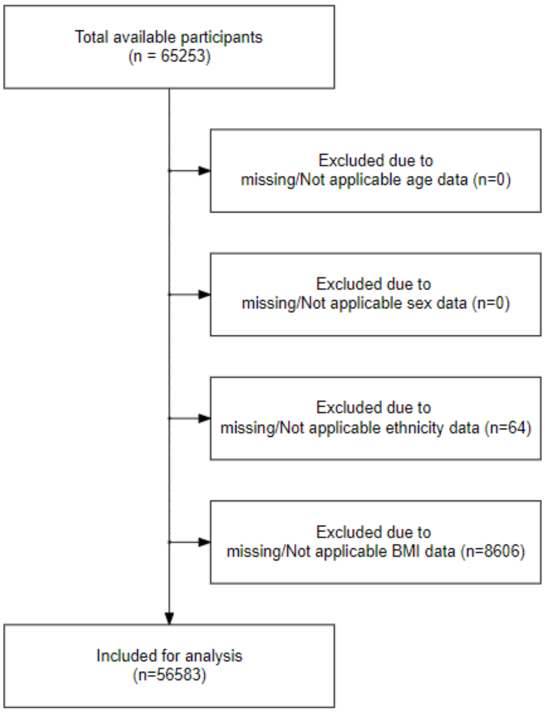

Appendix Table 16: Participants included for analysis for each of study from HSE data

Year	(n)
1995	3559
1996	3737
1997	6529
1998	3334
1999	2869
2000	1571
2001	3017
2002	6227
2003	2675
2004	1795
2005	1229
2006	2347

Year	(n)
2007	1127
2008	2283
2009	2418
2010	1260
2011	1213
2012	1189
2013	1344
2014	1211
2015	1234
2016	1130
2017	1096
2018	1080
2019	1109

Appendix IV Data Availability throughout study

Appendix Figure 9: Line graph demonstrating years in which data for each variable included for analysis was available throughout the study period.

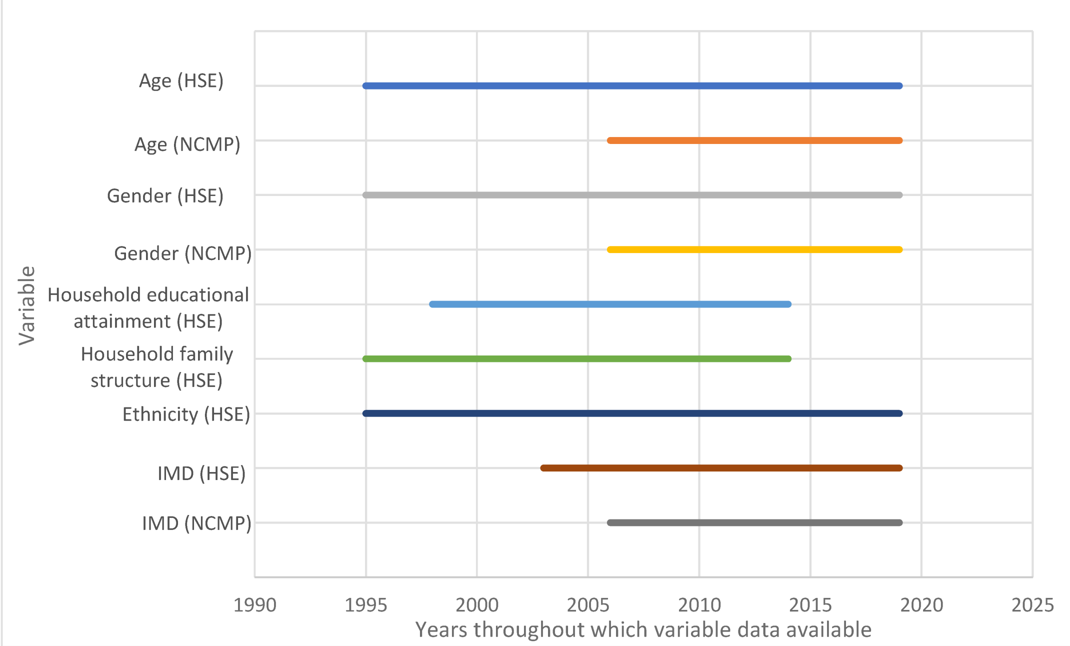

Variable	Start Year	End Year
Age (HSE)	1995	2019
Age (NCMP)	2006	2019
Gender (HSE)	1995	2019
Gender (NCMP)	2006	2019
Household educational attainment (HSE)	1998	2014
Household family structure (HSE)	1995	2014
Ethnicity (HSE)	1995	2019
IMD (HSE)	2003	2019
IMD (NCMP)	2006	2019
